# The Work of the R.A.M.C.: Another Record of Gallant Deeds

**Published:** 1915-09-25

**Authors:** 


### THE WORK OF THE R.A.M.C.

### Another Record of Gallant Deeds.

Sir Ian Hamilton and the R.A.M.C.

The columns of The Hospital have borne frequent tribute of late to the heroism of members of the R.A.M.C. engaged in the operations at the Dardanelles, and we are pleased to see that in his latest dispatch, issued this week, Sir Ian Hamilton expresses his appreciation of the way the medical service has been carried on in the face of "unusual and very trying conditions." "There are no roads," he writes, "and the wounded who are unable to walk must be carried from the firing line to the shore. They and their attendants may be shelled on their way to the beaches, at the beaches, on the jetties, and again—though, I believe, by inadvertence—on their way out in lighters to the hospital ships. Under shell fire it is not as easy as some of the critically-disposed seem to imagine to keep all arrangements in apple-pie order. Here I can only express my own opinion that efficiency, method, and even a certain quiet heroism have characterised the evacuations of the many thousands of our wounded."

Awards for Gallantry.

A supplement of the *London Gazette*, which has just been issued, contained a large number of awards by His Majesty for gallantry and devotion to duty on the part of his Army. Once again the Royal Army Medical Corps and ambulance workers figure prominently in the list of recipients, and the object of this article is to supplement the particulars of gallant deeds which have appeared in previous issues of The Hospital.

Conspicuous Bravery at Hooge.

Captain Kingsmill Williams Jones, M.D., R.A.M.C. (Special Reserve), who is attached to the 1st Battalion East Kent Regiment, wins the D.S.O. for conspicuous gallantry and devotion to duty at Hooge. During the entire night of August 9-10 and the whole of the following day and night he was attending to and evacuating wounded from the front trenches, time after time exposing himself to shell and rifle fire. He was twice slightly wounded, but stuck to his work with unflagging energy, and it was entirely owing to him that the crater was successfully evacuated of wounded. For conspicuous devotion to duty and energy on the same occasion the Military Cross goes to Temporary Lieutenant Thomas Lewis Ingram, of the R.A.M.C., and attached to the 1st Battalion Shropshire Light Infantry. It is recorded that he was evacuating wounded from the front trenches almost without cessation during the entire nights of August 9 and 10, and his indomitable energy and resource were the means of saving the lives of many severely wounded officers and men. The official report adds: "He has previously done consistently good work."

Gallantry at the Dardanelles.

A considerable proportion of the Distinguished Conduct Medals awarded to non-commissioned officers and men relate to gallantry during the Dardanelles fighting, and here the men of the Australian and New Zealand Field Ambulances have been conspicuous. For instance, Privates J. Comrie and W. J. Henry, of the New Zealand Field Ambulance, during and subsequent to the landing at Gaba Tepe attended on the wounded under a very heavy fire, allowing no danger to interfere with their duties, and they invariably showed the greatest courage and presence of mind. On the same occasion Lance-Corporal G. C. Farnham, of the 3rd Field Ambulance, Australian Imperial Force, showed the greatest zeal and disregard of danger in attending to the wounded under a heavy fire, and at all times he gave a fine exhibition of coolness and devotion to duty.

Striking indeed is the tribute paid by the *London Gazette* to Private E. P. Hitchcock and Private A. A. Morath, of the Australian Army Medical Corps, who are attached to the 6th Battalion of the Australian Imperial Force. Perhaps it had best speak for itself. It records that the two men are rewarded "for conspicuous gallantry and devotion to duty on May 8 and following days north of Cape Helles. In assisting the wounded under constant heavy fire they exhibited a heroism beyond praise. Absolutely regardless of danger, they attended to the wounded, leading up the stretcher-bearers and dressing the severe cases in the fire trenches, even before they were completed. Not only were they instrumental in saving many lives, but by their coolness and courage they set a splendid example of devotion to duty and gave the greatest encouragement to all ranks." Equally impressive is the tribute paid to Private J. V. F. Gregg-McGregor, of the 1st Field Ambulance, and Private C. H. G. Rosser, of the 3rd Field Ambulance of the Australian Imperial Force. After the landing at Gaba Tepe they "showed the greatest bravery and resource in attending to the wounded. Totally regardless of danger, they were for three consecutive days under a continuous and heavy shell and rifle fire, dressing and collecting the wounded from the most exposed positions. They allowed no personal risk or fatigue to interfere with the performance of their duties, and their gallant conduct and devotion offered a splendid example to all ranks."

On a Hospital Ship.

Private L. Crawford-Watson, of the New Zealand Army Medical Corps, gains his medal for gallant conduct and exceptionally good work in connection with the improvisation of the hospital ship *Lutzow*. He showed the greatest courage under all circumstances in attending to his duties, no work being too difficult or hazardous, and he gave a fine example of devotion to duty.

Sergeant J. W. Breame, of the 1st East Anglian Field Ambulance, R.A.M.C. (Territorial Force), displayed conspicuous gallantry on June 4 during the operations on the Gallipoli Peninsula. He showed great courage and zeal during the day in attending to and rescuing wounded under a heavy fire, allowing no personal risks to interfere with the performance of his work.

A Brave Ambulance Worker.

The awards to the men for gallant work during the terrible fire attacks at Hooge are not made on this occasion, though they may be expected shortly. The only ambulance worker rewarded in the present list for gallantry on the Western front is Private H. T. Cameron, of the No. 3 Field Ambulance of the 1st Canadian Division. In his case the record states that during the fighting at Festubert on the night of May 20-21 he was the first to volunteer to assist in collecting the wounded at the orchard captured from the enemy, which was still under a very heavy fire. The task was one of great difficulty and danger, and of the party of eight men who undertook it four were severely wounded.

